# Radiation dosimetry of [^18^F]-PSS232—a PET radioligand for imaging mGlu5 receptors in humans

**DOI:** 10.1186/s13550-019-0522-9

**Published:** 2019-06-25

**Authors:** Bert-Ram Sah, Michael Sommerauer, Linjing Mu, Gloria Pla Gonzalez, Susanne Geistlich, Valerie Treyer, Roger Schibli, Alfred Buck, Geoffrey Warnock, Simon M. Ametamey

**Affiliations:** 1 Department of Nuclear Medicine, University Hospital Zurich and University of Zurich, Zurich, Switzerland; 2 Department of Diagnostic, Interventional, and Pediatric Radiology, Inselspital, University of Bern, Bern, Switzerland; 3 Department of Neurology, University Hospital Zurich and University of Zurich, Zurich, Switzerland; 4 Radiopharmaceutical Science, Department of Chemistry and Applied Biosciences, Institute of Pharmaceutical Sciences, ETH Zurich, Zurich, Switzerland; 5 PMOD Technologies LLC, Zurich, Switzerland

**Keywords:** Dosimetry, Tracer, [^18^F]-PSS232, mGlu5 receptors

## Abstract

**Purpose:**

(E)-3-(pyridin-2-ylethynyl)cyclohex-2-enone O-(3-(2-[^18^F]-fluoroethoxy)propyl) oxime ([^18^F]-PSS232) is a new PET tracer for imaging of metabotropic glutamate receptor subtype 5 (mGlu5), and has shown promising results in rodents and humans. The aim of this study was to estimate the radiation dosimetry and biodistribution in humans, to assess dose-limiting organs, and to demonstrate safety and tolerability of [^18^F]-PSS232 in healthy volunteers.

**Methods:**

PET/CT scans of six healthy male volunteers (mean age 23.5 ± 1.7; 21–26 years) were obtained after intravenous administration of 243 ± 3 MBq of [^18^F]-PSS232. Serial whole-body (vertex to mid-thigh) PET scans were assessed at ten time points, up to 90 min after tracer injection. Calculation of tracer kinetics and cumulated organ activities were performed using PMOD 3.7 software. Dosimetry estimates were calculated using the OLINDA/EXM software.

**Results:**

Injection of [^18^F]-PSS232 was safe and well tolerated. Organs with highest absorbed doses were the gallbladder wall (0.2295 mGy/MBq), liver (0.0547 mGy/MBq), and the small intestine (0.0643 mGy/MBq). Mean effective dose was 3.72 ± 0.12 mSv/volunteer (range 3.61–3.96 mSv; 0.0153 mSv/MBq).

**Conclusion:**

[^18^F]-PSS232, a novel [^18^F]-labeled mGlu5 tracer, showed favorable dosimetry values. Additionally, the tracer was safe and well tolerated.

## Introduction

Positron emission tomography (PET) is a powerful imaging modality that enables imaging and quantitative measurement of tracer activity in vivo. [^18^F]-FDG is currently the most widely used tracer [1]. In spite of its high background and low selectivity in the brain, FDG is widely used in clinical routine in the diagnostic work up of neurodegenerative diseases [2, 3].

**Fig. 1:**
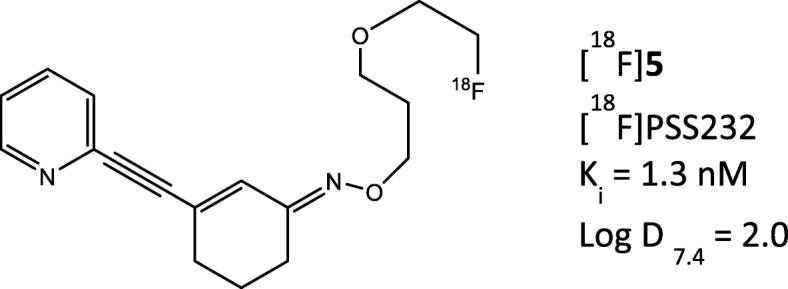
Chemical structure of [^18^F]-PSS232

In recent years, a plethora of receptor-selective brain tracers were developed and some of them have already entered the clinical arena [4–6]. An interesting target for brain studies is the metabotropic glutamate receptor (mGlu), a heterogeneous family of eight G-protein-coupled receptors, which are linked to multiple second messengers and modulation of ion channel activity in the central nervous system (CNS). The metabotropic glutamate receptor subtype 5 (mGlu5) is implicated in several brain disorders including schizophrenia, depression, anxiety, and Parkinson’s disease, in which [^18^F]-FDG is of limited use [7]. [^11^C]-ABP688 is a widely used PET radiotracer in the clinic and shows selective binding to mGlu5 receptors [7, 8]. The main limitation of [^11^C]-ABP688 is the short physical half-life of carbon-11 (20 min), which limits its wider application. For the purposes of centralized radiotracer production, a PET tracer labeled with fluorine-18 is desirable. Therefore, several fluorinated analogs of [^11^C]-ABP688 were investigated. [^18^F]-PSS232 (Fig. 1) showed favorable in vitro and in vivo properties and high selectivity for mGlu5 [9, 10]; therefore, it was selected for translation into humans.

The aim of this study was to evaluate the radiation dosimetry of [^18^F]-PSS232 and to determine its safety and tolerability in healthy volunteers.

## Materials and methods

### Ethics approval, consent to participate, and volunteers

All procedures performed in studies involving human participants were in accordance with the ethical standards of the institutional and/or national research committee and with the 1964 Helsinki declaration and its later amendments or comparable ethical standards. The institutional review board approved this study (KEK-ZH Nr. 2013-0100). Six healthy male volunteers were prospectively selected for the study and gave written informed consent prior to inclusion into the study.

### Preparation of 18F-PSS232

The radiosynthesis of [^18^F]-PSS232 was performed as reported previously [9]. Briefly, [^18^F]-PSS232 was prepared via aliphatic nucleophilic substitution by reacting the mesylate precursor with [^18^F]fluoride in the presence of Kryptofix-222® in anhydrous dimethylsulfoxide at 95 °C for 10 min. After HPLC purification, the product was collected and trapped on a Light C18 cartridge and eluted with EtOH (1.5 mL) in a sterile vial containing 16.5 mL saline and 450 mg sodium ascorbate. The radiolabeled product was confirmed by co-elution with unlabeled PSS232. Molar radioactivity ranged from 70 to 150 GBq/μmol at end of the synthesis, and purity was ≥ 98%.

### Safety monitoring

Vital signs (blood pressure, body temperature, and heart rate) and adverse effects were assessed in all patients.

### Data acquisition

After bolus injection of 243.4 ± 2.6 MBq of [^18^F]-PSS232 into a cubital or antebrachial vein, all volunteers underwent standardized serial whole-body PET/CT imaging (vertex of skull to mid-thigh) using an integrated PET/CT system (DiscoveryTM VCT; GE Healthcare, Milwaukee, Wisconsin, USA), which is under routine maintenance and cleared for clinical use. Subjects were encouraged to hydrate well and void their bladder before and after the scan. They were asked to lie on the examination table for the entire acquisition. In total, ten emission scans were acquired at time points 0, 10, 20, 30, 40, 50, 60, 70, 80, and 90 min after injection of [^18^F]-PSS232. Scan duration at each time point was 6 min. Before the beginning of the serial PET series, a standard low-dose CT was performed for attenuation correction of the PET scan and assistance in organ delineation.

### Data analysis

The ten emission scans were merged into a dynamic series and tracer kinetics were quantified using the coregistered dynamic PET and CT (PMOD 3.7 Fusion, PMOD Technologies LLC, Zurich, Switzerland). Volumes of interest (VOIs) were placed in the brain, thyroid, thymus, heart wall, heart content, liver, gall bladder, small intestine, pancreas, kidneys, spleen, muscles, urinary bladder, and bone marrow (proximal humerus). It was taken care to delineate whole organs. This was done manually and visually by an experienced nuclear physician and radiologist, considering the anatomical edge as shown in the CT scan, as well as manually adjusting the VOIs in each PET scan separately. For bone marrow, we delineated the proximal humerus, and scaled that kBq/mL average to the “standard human” full organ mass as given by OLINDA. There is no information available about specific binding of the tracer to any components of the bone marrow.

The x-axis for each time activity curve was adjusted to account for the time difference between individual PET fields-of-view in construction of whole body images. Analysis of the PET-derived organ time-activity curve (TACs) to Bq, incorporating radioactive decay, was performed automatically in PMOD (Kinetic Modeling module). Following conversion, the cumulated organ activity (Bq-hr/Bq) was calculated using trapezoidal integration for the duration of the PET data, and analytical integration of the decay to infinity from the end of the PET data. Total organ cumulated activity was calculated using the standard organ volumes reported in the OLINDA/EXM documentation. The remainder fraction was calculated by subtracting the summed organ residence times from the radionuclide theoretical whole body residence time, which for [^18^F] is 2.6401 h [11]. Effective dose, and individual organ doses, was calculated for each patient using OLINDA/EXM Version 1.0 (Version OLINDA/EXM 1.0, Vanderbilt University, Nashville, TN, USA). ICRP 60 method was used. No bladder-voiding model was used because of the lipophilic structure and dominantly excretion through the biliary system. ICRP 30 gastrointestinal model was used for simulation of 15% of liver and 50% of gall bladder activity entering the small intestine.

## Results

### Safety

The injection of 243.4 ± 2.6 (range 240.5–247.2) MBq [^18^F]-PSS232 was well tolerated. An overview over the injected dose of [^18^F]-PSS232 is shown in Table 1. Mean body temperatures during screening, before injection of tracer, and after imaging were 36.6 °C (range 35.8–37.2), 36.6 °C (range 36.0–37.2), and 36.6 °C (36.2–37.1), respectively. Mean systolic blood pressures during screening, before injection of tracer, and after imaging were 120.3 mmHg (range 107–130), 125.2 mmHg (range 95–140), and 113.2 mmHg (range 95–123), respectively. Mean diastolic blood pressures during screening, before injection of tracer, and after imaging were 66.5 mmHg (range 59–77), 74.7 mmHg (range 51–102), and 66.3 mmHg (range 58–79), respectively. Mean heart rates at screening, before injection of tracer, and after imaging were 58 (range 43–86), 61 (range 43–80), and 66 (range 53–72), respectively. None of the volunteers reported any discomfort with regards to the injection.

**Table 1:** Subject characteristics and injected radioactivity of [^18^F]-PSS232

Volunteer	Sex	Age (years)	Injected dose (MBq)	Effective dose (mSv/patient)
1	M	25	243.2	3.96
2	M	21	240.5	3.66
3	M	22	240.6	3.73
4	M	24	246.6	3.65
5	M	23	247.2	3.73
6	M	26	242.4	3.61
Mean ± SD		23 ± 1.7	243.4 ± 2.6	3.72 ± 0.12

### Radiation dosimetry estimates and biodistribution

**Fig. 2:**
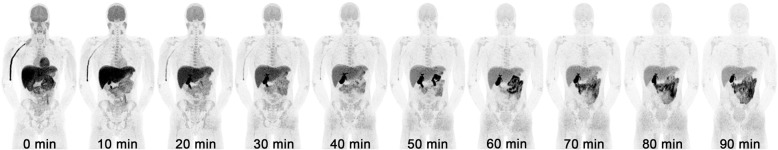
Maximum-intensity projection PET images of the sequential whole body PET-scans from 0 to 90 min after injection of [^18^F]-PSS232 in the right cubital vein. All images are displayed with the same intensity scale

**Fig. 3:**
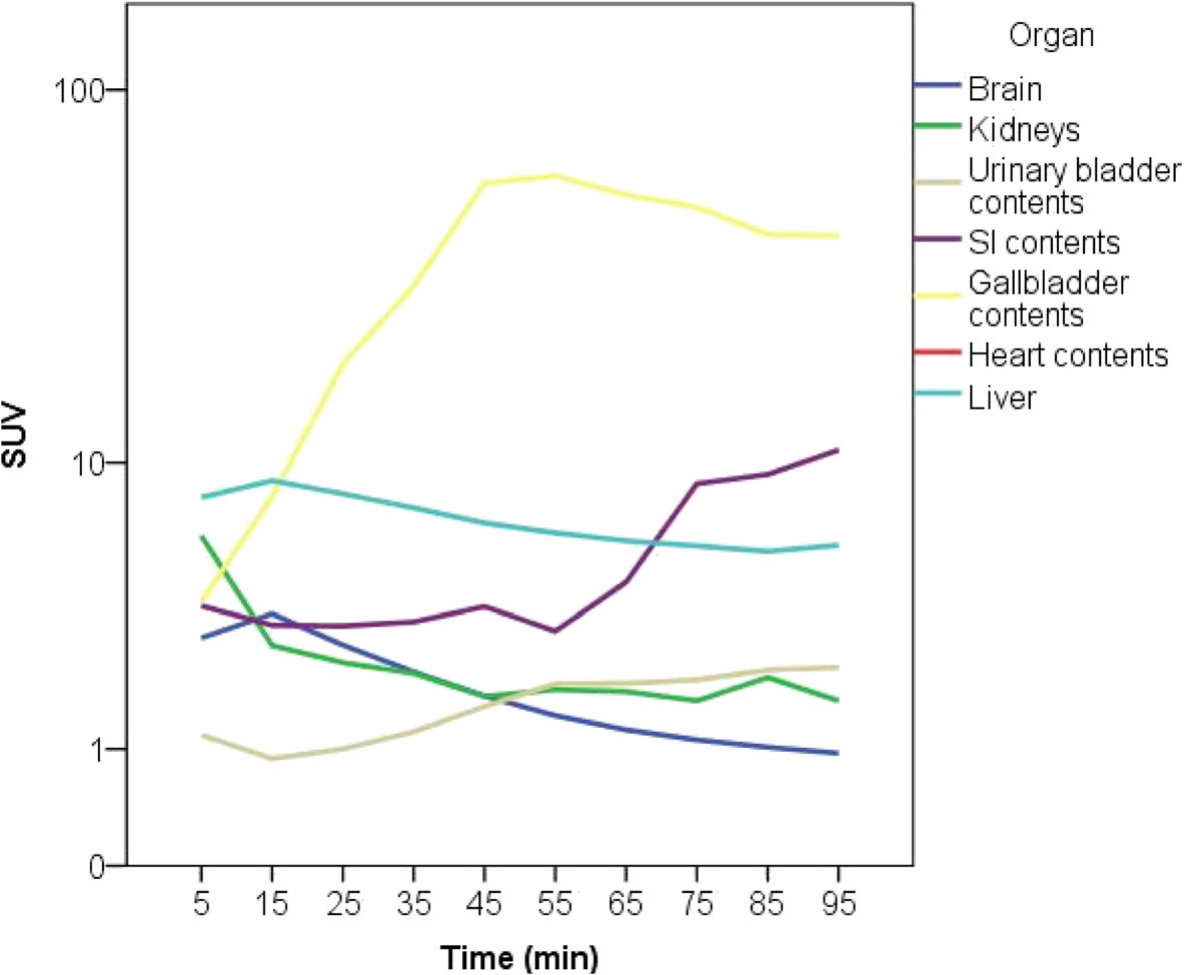
Typical time–activity curves for selected organs (liver, gallbladder wall, small intestine, brain, kidneys, and urinary bladder after injection of 240.5 MBq [^18^F]-PSS232)

An example of a typical serial whole-body PET (maximum intensity projection) is presented in Fig. 2. Typical time-activity curves of representative organs are shown in Fig. 3. Cumulated organ activities are shown in Table 2. Activity was secreted through the liver into the bile. Thus, the highest radiation dose was received by the gallbladder wall (0.2295 mGy/MBq), liver (0.0547 mGy/MBq), and the small intestine (0.0643 mGy/MBq). Mean effective dose was 3.72 ± 0.12 mSv/volunteer (range 3.61–3.96 mSv). All calculated radiation dose estimates are summarized in Table 3, and results using ICRP 30 gastrointestinal model in Table 4.

**Table 2:** Mean organ residence times. Data is presented as Bq-hr/Bq administered (mean ± SD; *n* = 6)

Source organ	Mean residence time
Brain	0.0527 ± 0.0066
Gallbladder contents	0.1348 ± 0.0811
Small intestine	0.2702 ± 0.0499
Heart contents	0.0096 ± 0.0018
Heart wall	0.0109 ± 0.0017
Kidneys	0.0174 ± 0.0024
Liver	0.3873 ± 0.0716
Muscle	0.7552 ± 0.0174
Pancreas	0.0039 ± 0.0009
Red marrow	0.0681 ± 0.0137
Spleen	0.0035 ± 0.0014
Thymus	0.0008 ± 0.0002
Thyroid	0.0005 ± 0.0001
Urinary bladder contents	0.0200 ± 0.0181
Remainder	0.9050 ± 0.3442

**Table 3:** Absorbed organ dose (mGy/MBq) and effective dose (mSv/MBq) after injection of [^18^F]-PSS232. Coefficients of variation (COV) (ratio of SD to mean)

Organ	Mean	COV	Minimum	Maximum
Adrenals	0.0138	2.2	0.0134	0.0143
Brain	0.0107	10.3	0.0089	0.0121
Breasts	0.0064	17.2	0.0050	0.0080
Gallbladder wall	0.2295	55.4	0.0567	0.4340
Lower large intestine wall	0.0130	7.7	0.0115	0.0145
Small intestine	0.0643	14.6	0.0510	0.0816
Stomach wall	0.0118	5.1	0.0111	0.0128
Upper large intestine wall	0.0217	6	0.0207	0.0246
Heart wall	0.0153	9.2	0.0129	0.0172
Kidneys	0.0209	11	0.0177	0.0249
Liver	0.0547	18.1	0.0440	0.0727
Lungs	0.0090	7.8	0.0081	0.0101
Muscle	0.0108	8.3	0.0093	0.0119
Ovaries	0.0156	6.4	0.0138	0.0169
Pancreas	0.0197	12.2	0.0175	0.0246
Red marrow	0.0140	4.3	0.0129	0.0145
Osteogenic cells	0.0136	11.8	0.0113	0.0154
Skin	0.0059	13.6	0.0048	0.0071
Spleen	0.0105	14.3	0.0092	0.0135
Testes	0.0071	14.1	0.0054	0.0088
Thymus	0.0121	10.7	0.0104	0.0147
Thyroid	0.0088	6.8	0.0077	0.0096
Urinary bladder wall	0.0188	43.1	0.0140	0.0368
Uterus	0.0151	4.6	0.0142	0.0164
Total body	0.0123	0.8	0.0121	0.0123
Effective dose	0.0153	3.3	0.0148	0.0163

**Table 4:** Absorbed organ dose (mGy/MBq) and effective dose (mSv/MBq) using ICRP 30 gastrointestinal model

Organ	Mean	COV	Minimum	Maximum
Adrenals	0.0129	2.3	0.0125	0.0133
Brain	0.0107	10.3	0.0089	0.0121
Breasts	0.0063	17.5	0.0048	0.0080
Gallbladder wall	0.1368	57.5	0.0356	0.2780
Lower large intestine wall	0.0200	14.5	0.0151	0.0250
Small intestine	0.0422	24.2	0.0256	0.0600
Stomach wall	0.0112	3.6	0.0108	0.0117
Upper large intestine wall	0.0473	25.8	0.0274	0.0685
Heart wall	0.0149	8.7	0.0126	0.0166
Kidneys	0.0199	11.6	0.0165	0.0236
Liver	0.0493	20.1	0.0372	0.0648
Lungs	0.0087	8.0	0.0077	0.0099
Muscle	0.0105	9.5	0.0087	0.0117
Ovaries	0.0146	6.2	0.0130	0.0159
Pancreas	0.0183	11.5	0.0162	0.0225
Red marrow	0.0136	5.1	0.0120	0.0143
Osteogenic cells	0.0134	11.2	0.0111	0.0151
Skin	0.0058	13.8	0.0046	0.0069
Spleen	0.0101	14.9	0.0088	0.0132
Testes	0.0071	14.1	0.0055	0.0087
Thymus	0.0120	10.8	0.0103	0.0146
Thyroid	0.0088	6.8	0.0077	0.0096
Urinary bladder wall	0.0185	44.3	0.0137	0.0369
Uterus	0.0134	3.0	0.0128	0.0140
Total body	0.0117	1.7	0.0113	0.0119
Effective dose	0.0150	8.7	0.0131	0.0170

## Discussion

Since the identification of mGlu5 as a promising target, a plethora of radiolabeled tracers have been evaluated preclinically [8, 12–15]. Only a few have progressed to the clinical setting [16–19]. Recently, [^18^F]-PSS232 was identified as a new fluorinated derivative, which can be reliably prepared in high radiochemical yields and with high molar radioactivity. It was demonstrated to readily cross the blood–brain barrier and to selectively bind to mGlu5-rich regions in the rat brain [9, 10]. In order to further investigate this promising tracer, an exploratory clinical trial was performed.

PET imaging with [^18^F]-PSS232 was shown to be safe and well tolerated in all volunteers. No adverse events were observed in any studied subjects. The effective dose of 0.0153 mSv/MBq is favorable and in the same range as the reported dose of a recently published fluorine-18-labeled mGlu5 tracer ([^18^F]-FPEB; 0.0149–0.0250 mSv/MBq, depending on bladder voiding model [19]). Among the six volunteers, the effective dose showed very low inter-individual variability (SD = 0.0005). Radiation exposure was lower than the reported dose for clinically used fluorine-18-labeled tracers such as fluoromethylcholine (0.031 mSv/MBq) [20] and comparable to the dose of [^18^F]-fluoroethyl-tyrosine (0.016 mSv/MBq) [21]. [^18^F]-FPEB is more lipophilic than [^18^F]-PSS232; [^18^F]-PSS232 exhibits a logD value of 2.0, whereas [^18^F]-FPEB shows a value of 2.8.

Due to the lipophilic properties of the tracer, it is primarily excreted through the hepatobiliary system, and consequently the highest radiation doses are received by the gallbladder wall, the small intestine, and the liver. Our results overestimate the dose received by the gallbladder wall and the liver due to incomplete clearance of the tracer from the liver parenchyma at the end of the imaging study, whereas for the calculation of cumulated organ activities we assumed all activity to remain in the organ from that point of time onwards. This predominantly influences the gallbladder and small intestine (Fig. 2) cumulated organ activities. Encouraging patients to consume a fatty meal after imaging might reduce the activity received by the gallbladder wall significantly, since tracer secretion with the bile into the small intestine could be accelerated. However, this has to be proven in another study. Cumulated organ activity of the gall bladder might be overestimated, and of the large bowel underestimated, because it can be expected that tracer is cleared from small to large bowel within hours (Table 4 for results using ICRP 30 gastrointestinal model). Beyond that, organ doses in our study are in the same range as other lipophilic tracers [18]. The dosimetry results for [^18^F]-PSS232 confirmed a low radiation dose to the urinary system (Table 3). As expected for a lipophilic tracer, this is significantly lower than reported doses for [^18^F]-FDG.

## Conclusion

[^18^F]-PSS232 is a well-tolerated imaging probe for mGlu5. Due to its high lipophilicity, the tracer is excreted through the hepatobiliary system. It shows favorable dosimetry in humans, opening the possibility for further studies in patients.

## Abbreviations

[^18^F]-PSS232: (E)-3-(pyridin-2-ylethynyl)cyclohex-2-enone O-(3-(2-[^18^F]-fluoroethoxy)propyl) oxime
CNS: Central nervous system
mGlu5: Metabotropic glutamate receptor subtype 5
PET: Positron emission tomography
TAC: Time-activity curve
